# Investigating the causal effect of previously reported therapeutic agents for colorectal cancer prevention: protocol for a Mendelian randomization analysis

**DOI:** 10.12688/wellcomeopenres.20861.2

**Published:** 2024-06-13

**Authors:** Ella Fryer, Richard M. Martin, Philip Haycock, James Yarmolinsky

**Affiliations:** 1 MRC Integrative Epidemiology Unit, University of Bristol, Bristol, England, BS8 2BN, UK; 2 Population Health Sciences, University of Bristol, Bristol, England, BS8 2BN, UK; 3 NIHR Bristol Biomedical Research Centre, University Hospitals Bristol and Weston NHS Foundation Trust, University of Bristol, Bristol, England, BS8 2BN, UK; 4 Department of Epidemiology and Biostatistics, School of Public Health, Imperial College London, London, England, W2 1PG, UK

**Keywords:** Preventive therapy, Chemoprevention, Colorectal cancer, Mendelian randomization

## Abstract

**Background:**

Colorectal cancer (CRC) is the third most common cancer worldwide, with 1.9 million new cases in 2020 and a predicted rise to 3.2 million in 2040. Screening programmes are already in place to aid early detection and secondary prevention of CRC, but the rising prevalence means additional approaches are required in both primary and secondary prevention settings. Preventive therapy, whereby natural or synthetic agents are used to prevent, reverse or delay disease development, could be an effective strategy to further reduce cancer risk and potential agents have already been identified in conventional observational studies. However, as such studies are vulnerable to confounding and reverse causation, we aim to evaluate these observed relationships using Mendelian randomization (MR), an alternative causal inference approach which should be less susceptible to these biases.

**Methods and analysis:**

We will use two-sample MR, which uses two independent samples for the exposure and outcome data, to investigate previously reported observational associations of multiple potential preventive agents with CRC risk. We define preventive agents as any synthetic (e.g. approved medication) or natural (e.g. micronutrient, endogenous hormone) molecule used to reduce the risk of cancer. We will first extract potential preventive agents that have been previously linked to CRC risk in observational studies from reviews of the literature. We will then evaluate whether we can develop a genetic instrument for each preventive agent from previously published genome-wide association studies (GWASs) of direct measures of molecular traits (e.g. circulating levels of protein drug targets, blood-based biomarkers of dietary vitamins). The summary statistics from these GWASs, and a large GWAS of CRC, will be used in two-sample MR analyses to investigate the causal effect of putative preventive therapy agents on CRC risk. Sensitivity analyses will be conducted to evaluate the robustness of findings to potential violations of MR assumptions.

## Plain Language Summary

Colorectal cancer is the third most common cancer worldwide and the second most common cause of cancer-related death. An individual’s chances of surviving the disease are increased if it is caught early or even prevented from developing in the first place. Currently, screening in the UK is offered to everyone over the age of 50 and whilst this can be effective in early cancer detection and secondary prevention, additional prevention strategies are needed to reduce cancer rates.

Previous research has investigated whether intake of certain medications, dietary micronutrients or hormones can help prevent colorectal cancer development. There is some evidence to show that taking aspirin can reduce your risk of developing colorectal cancer, however, given potential adverse side effects to taking aspirin (e.g. gastrointestinal bleeding), this medication is not suitable for everyone. We wanted to assess whether other previously identified medications, micronutrients or hormones have any effect on reducing risk of cancer development. If we can identify a compound that could reduce an individual’s risk of developing colorectal cancer, it could be used as an additional cancer prevention strategy.

We curated a list of potential preventive compounds from previously conducted studies that used observational epidemiological (i.e. non-randomised) methods. Distinguishing statistical correlations from causal relationships when using observational methods can be difficult. This can be due to additional factors in the study affecting both the use of potential preventive compounds and an individual’s risk of cancer development (e.g. those taking vitamin supplements having a healthier diet) or the observed relationship may be causal but in the opposite direction (e.g. a cancer diagnosis has a subsequent effect on dietary habits). We will use an alternative epidemiological method, called Mendelian randomization, which uses genetics to attempt to overcome this issue and enable us to determine if a specific compound is reducing cancer risk.

## Introduction

Colorectal cancer (CRC) is a major public health issue, being the third most common cancer globally, with an estimated 1.9 million new cases in 2020 ^1– 3^. A small proportion of cases are attributable to rare, highly penetrant germline mutations; however, the majority of cases are believed to be influenced by a combination of genetic, environmental and lifestyle factors. In many countries cases have been increasing in line with economic growth, given the accompanied elevated exposure to CRC risk factors (e.g. poor diet, lack of exercise, ageing population), and this has led to estimations of new cases reaching 3.2 million in 2040 ^4, 5^. While in some high-income countries incidence rates have remained relatively stable or even decreased over the last two decades (largely attributable to screening programmes), incidence rates among a younger demographic (i.e., under the age of 50) have increased over this time period ^6– 8^.

Prevention is a key strategy for reducing disease burden, and there are a number of approaches that could be used to intervene at different stages of cancer initiation and progression ^9^. Primary prevention, where an intervention is deployed in a healthy population (e.g. showing no signs of disease or precursors), can either be employed universally or targeted at higher risk groups (e.g. individuals with Lynch syndrome). Examples of primary prevention strategies include lifestyle interventions promoting smoking cessation and weight loss and pharmacological approaches like aspirin use ^10^. Secondary prevention refers to detection of the early stages of, or the precursor to (e.g. polyps for CRC), the disease and intervening before the disease progresses. Screening of CRC is an example of secondary prevention and has been found to reduce cancer incidence and mortality in average-risk groups ^11^, through enabling early detection and subsequent removal of pre-cancerous growths ^4, 7^. Screening alone is insufficient for reducing disease burden and additional strategies are needed for both primary and secondary prevention, in both the general population and in high-risk groups.

Preventive pharmacological therapy is one strategy that could be employed for CRC prevention, whereby those in the general population or at high risk of CRC would take a therapeutic agent over an extended period of time, consequently reducing their lifetime risk of developing the disease ^12^. Preventive therapy agents can be synthetic or natural molecules, such as drugs, dietary micronutrients and endogenous hormones. The biological effect of a preventive agent is often to increase or decrease the concentrations of certain molecules in the blood, which will have downstream effects that can lead to a reduction in cancer development ^13^. Conventional observational studies have reported associations between various preventive agents and CRC risk reduction, which has subsequently informed the testing of certain agents in large-scale clinical trials. For example, conventional observational studies suggesting a protective role of aspirin in CRC development ^14, 15^ informed design of large-scale randomised controlled trials which have corroborated these findings, supporting aspirin as a preventive agent for CRC ^16– 18^. There are risks associated with aspirin use, the most frequently reported relating to gastrointestinal toxicity, and this has been observed with both regular and low-dose use ^19^. There is therefore a need to discover other potential preventive agents, with improved safety profiles, that are appropriate for long-term use for the purposes of primary or secondary CRC prevention.

Associations of preventive agents with CRC risk identified from observational evidence are susceptible to biases such as confounding (unmeasured and residual) and reverse causation. Consequently, it is often unclear whether associations identified in these studies represent causal relationships. An alternative epidemiological approach that can be employed to evaluate causal relationships in observational settings is Mendelian randomization (MR), which uses germline genetic variants to instrument exposures to minimise these sources of bias, strengthening causal inference ^20– 23^. As alleles of germline genetic variants are randomly assorted at meiosis and fixed from conception, conventional sources of confounding and reverse causation should be minimised.

In order to conduct MR studies, genetic instruments are required that are robustly associated with the exposure of interest. There has been a proliferation of genome-wide association studies (GWASs) that have identified associations between large numbers of traits and germline variants across the genome. These include direct measurements of molecular traits (e.g. levels of protein targets of drugs, circulating dietary biomarkers and endogenous hormones), making it possible to evaluate causal relationships between molecular exposures and outcomes in a two-sample MR framework.

## Aims

The aim of this study is to use two-sample Mendelian randomization to estimate the effect of various previously reported preventive therapies on CRC risk.

## Methods

### Study design

Two-sample MR will be used to investigate the causal relationship between putative preventive therapy agents and risk of CRC. The preventive agents investigated in these analyses will include medications, dietary micronutrients and endogenous hormones. We are prioritising these types of agents as they can be modified through either supplementation (e.g. increasing vitamin levels) or pharmacological perturbation (e.g. inhibiting protein drug targets). For previously reported medications, we will attempt to instrument the target of these medications (e.g. circulating protein targets). For previously reported dietary micronutrients, we will instrument circulating measures of these micronutrients (e.g. blood-based biomarkers for dietary vitamins). For circulating endogenous hormones, these will be instrumented directly. For simplicity we will collectively refer to the different instrument classes (e.g. circulating targets of medications, circulating dietary biomarkers, and circulating endogenous hormones) as molecular traits. Genetic instruments for each molecular trait will be constructed using single nucleotide polymorphisms (SNPs) associated with these molecular traits, obtained from summary statistics of genome-wide association studies (GWASs). We will then extract these SNPs from an independent GWAS of CRC risk to estimate causal effects. The Strengthening the Reporting of Observational Studies in Epidemiology using Mendelian Randomization (STROBE-MR) guidelines (up to and including the methods section) ^24^ were used to structure the writing of this protocol.

### Exposure data and instrument selection

A literature search was conducted to identify preventive agents that have previously been linked to reduced CRC risk in conventional observational epidemiological studies. PubMed and EMBASE were searched to identify reviews published from 1 ^st^ January 2013 to 7 ^th^ November 2023 ( Table 1). After screening, 17 reviews were included and, for each of these, all reported preventive agents were extracted.

**Table 1.** Search terms for literature review of putative preventive agents and CRC risk.

Database	Search terms	No. results
Pubmed	#1 "Colorectal Neoplasms"[MeSH Terms:noexp] OR "Rectal Neoplasms"[MeSH Terms:noexp] OR "bowel cancer"[Title/Abstract]	161,351
	#2 Chemoprevention"[MeSH Terms:noexp] OR "preventive therapy"[Title/Abstract]	10377
	#3 "Review"[Publication Type:noexp] OR "Review"[Title/Abstract]	4,062,927
	#4 "2013/01/01"[Date - Publication] : "2023/11/07"[Date - Publication]	13,813,521
	#5 #1 AND #2 AND #3 AND #4	64
Embase	1 exp colorectal cancer/	392,588
	2 exp colon tumor/	172,044
	3 exp rectum tumor/	78,359
	4 chemoprevention.ab,ti.	14,943
	5 preventive therapy.ab,ti.	5,085
	6 exp "review"/	3,200,578
	7 review.ab,ti.	2,714,988
	8 1 or 2 or 3	441,101
	9 4 or 5	19,979
	10 6 or 7	4,564,148
	11 8 and 9 and 10	915
	12 limit 11 to dc=20130101-20231107	338

*Abbreviations: CRC = colorectal cancer; MeSH = Medical Subject Heading; noexp = no explosion; ab = abstract; ti = title. Search terms used to search PubMed and Embase for reviews of observational studies that had associated preventive therapies with CRC risk, showing number of results returned at each step.*

For all preventive agents identified in the literature search, their effect on CRC risk will be tested. We will attempt to instrument the corresponding molecular trait for each agent. If the preventive agent is a drug, the molecular trait we will be testing will be the biological target of the drug which we will identify using the online databases Open Targets ^25^ and DrugBank ^26^. There may be challenges in designing instruments for some molecular traits including limited understanding of the mechanism of action of a drug (e.g. metformin) or the absence of genetic variants that are strongly associated with a trait (e.g. reaching genome-wide significance of p<5°10 ^-8^). Consequently, these molecular traits will be excluded from subsequent analyses.

For those molecular traits that will be taken forward for further analyses, genetic instruments will be constructed by identifying GWASs that have reported genetic associations with direct measures of molecular traits of interest in populations of European ancestry. These studies, and available instruments, will be searched for using several databases. Firstly, the GWAS catalogue ^27^ and the IEU Open GWAS ^28^ will be searched, followed by PubMed and medRxiv/bioRxiv for any pre-prints. We will also consult in-house summary genetic association statistics, if necessary, to access restricted and unpublished datasets. Genetic instruments will be constructed by selecting SNPs strongly associated with each trait of interest (p<5°10 ^-8^) using summary genetic association data from these studies. For those molecular traits that are protein drug targets or biomarkers, SNPs will be selected that are located in or within proximity to genes that code for that protein (termed *cis*-variants). *Cis*-variants are expected to be specific to the target being instrumented which should minimise the likelihood of bias from horizontal pleiotropy. In addition, the use of *cis*-acting variants that influence protein targets of drugs examined would be expected to enhance instrument strength because protein expression is relatively proximal to germline genetic variants. In the absence of SNPs associated with direct measures of protein targets, we will explore constructing instruments from SNPs associated with downstream biomarker perturbations of drug targets. For those molecular traits that correspond to dietary agents or endogenous hormones, genome-wide significant variants will be selected independent of their genomic position. Of these variants, only those that are independent (r ^2^<0.001) will be included as instrumental variables in the primary analyses. For protein drug targets and biomarkers, we will increase the number of SNPs in our genetic instrument by permitting SNPs to be in weak linkage disequilibrium (e.g. r ^2^<0.10). We will account for this by generating a correlation matrix using a random subset of 10,000 individuals of white British ancestry in the UK Biobank as reference panel and using a generalised IVW method that can account for correlated instruments ^29^.

### Outcome data

Genetic association data for CRC risk will be obtained from a GWAS meta-analysis of colorectal cancer in 78,473 cases and 107,143 controls ^30^. This study used data from several studies of individuals of European ancestry, including the Genetics and Epidemiology of Colorectal Cancer Consortium (GECCO), the Colorectal Transdisciplinary Study (CORECT), COloRectal cancer Study of Austria (CORSA) and UK Biobank. This GWAS was adjusted for age, sex and genetic principal components of ancestry (PCs). Details on participants, genotyping and quality control (QC) have been described previously ^30^. The summary data from this GWAS is available without UK Biobank data. Where there is sample overlap between the CRC GWAS and the exposure GWAS (i.e. those studies conducted exclusively in UK Biobank) and we have a significant finding from the main analyses after accounting for multiple testing (using a false discovery rate (FDR) correction of <5%), we will conduct a sensitivity analysis using CRC data with UK Biobank excluded. We will also compare the individual studies included in the CRC GWAS with all exposure datasets (i.e. beyond overlap of UK Biobank participants). Where there is a small-to-modest proportion of participant overlap between the studies included in the exposure data and the CRC data, we will quantify this overlap and, for any significant findings, we will repeat analyses using a more stringent F-statistic threshold (e.g. 31) to select genetic instruments. Sample overlap is only an issue if the genetic instruments used are weak ^32, 33^, therefore repeating analyses with a more stringent F-statistic threshold should minimise the likelihood of including weak instruments in analyses.

### Power

**Figure 1.**
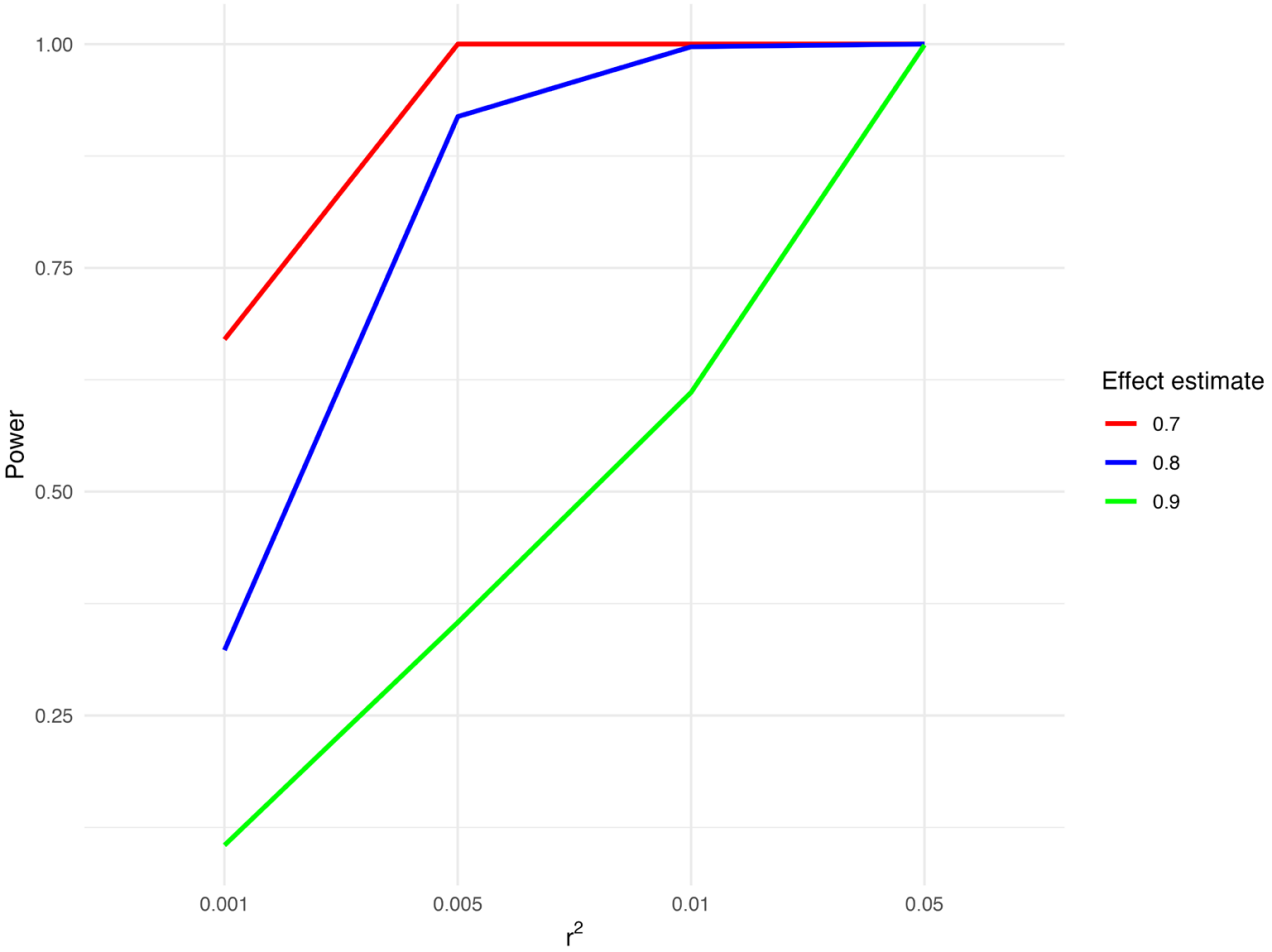
Potential statistical power to detect an association between preventive therapy agents and CRC risk. Abbreviations: CRC = colorectal cancer. Statistical power to detect an association between preventive therapy agents and CRC risk was calculated for three effect estimates (odds ratio per standard deviation increase in risk factor) (0.7, 0.8, 0.9) and a range of r ^2^ values (0.001, 0.005, 0.01, 0.05) with 5% significance level and total sample size of 185616 (cases=78473, controls=107143).

As we will be testing the effect of a number of different molecular traits on CRC risk, the phenotypic variance explained by the instruments (r ^2^) used for each trait will vary. We have therefore calculated ^31^ the statistical power of analyses for a range of r ^2^ values and odds ratios ( Figure 1).

### Data harmonisation

The summary statistics from the exposure and outcome data will be harmonised so that the effect allele of a particular SNP in the exposure dataset corresponds to the same allele of that same SNP in the outcome dataset. If alleles need to be switched in the outcome dataset, the effect estimate for the outcome will also be changed to reflect this. SNPs included in the harmonised data must therefore be present in both the exposure and outcome datasets.

Harmonisation will be carried out using the TwoSampleMR package in R (v0.5.8) ^34^. The harmonise_data function will be used with the default setting which uses allele frequencies to resolve palindromic SNPs (e.g. where the effect/non-effect allele are either A/T or G/C combinations). If it is not possible to resolve strand ambiguity for palindromic SNPs (i.e. the minor allele frequency is above 0.42) these SNPs will be excluded from subsequent analyses. To check that the harmonisation step has worked as expected, the correlation between effect allele frequencies in the exposure and outcome dataset will be calculated. A correlation close to 1 would indicate the data were successfully harmonised (and would also indicate that the exposure and outcome datasets are representative of the same underlying population, a key assumption of the two-sample MR approach).

### Mendelian randomization

***Assumptions*.** To provide a valid test of the causal null hypothesis, MR requires three instrumental variable (IV) assumptions to hold: “relevance”, “exchangeability”, “exclusion restriction” ^20– 23^. The “relevance” assumption states that the instrumental variable is strongly associated with the exposure of interest (e.g. a particular molecular trait being evaluated). The “exchangeability” assumption states there are no confounders (e.g. population structure) of the instrument and outcome. The “exclusion restriction” assumption states that the instrumental variable must only be associated with the outcome through the exposure (e.g. there is no horizontal pleiotropy, where the instrument influences the outcome through pathways independent of the exposure). Under the assumption of monotonicity, valid point estimates can be generated for those participants whose exposure is influenced by the instrument (i.e. a local average treatment effect).

### Main analyses

The proportion of variance in the molecular traits explained by the SNPs used as instrumental variables (r ^2^) and the strength of the instrument (F-statistic) will be calculated. The F-statistic can be used to test the “relevance” assumption and a threshold of >10 indicates that weak instrument bias is unlikely ^35^.

The proportion of variance explained in the instruments (R2) will be calculated using the following formula:

$$R^{2}=\frac{2\beta^{2}MAF(1-MAF)}{2\beta^{2}MAF(1-MAF)+{\left(se(\beta )\right)}^{2}(2N)MAF(1-MAF)}$$

Where β is the effect size (beta coefficient) for a given SNP, MAF is the minor allele frequency, se(β) is the standard error of the effect size, and N is the sample size. 

F-statistics will be calculated using the following formula:

$$F=R^{2}(N-2)/(1-R^{2})$$

Where R2 is the proportion of variance explained in the instrument, N is the sample size

For molecular traits instrumented by a single SNP, the Wald ratio will be used to calculate the effect of the molecular exposure on CRC risk ^23^. For those traits that are instrumented by more than one SNP, the inverse-variance weighted (IVW) method will be used ^23^. The multiplicative random effects IVW will be prioritised, except where the number of SNPs is limited (e.g. ≤3) in which case the fixed effects IVW will be used.

To account for multiple testing, we will use a false discovery rate (FDR) correction of <5% to define strong evidence.

We will attempt to explore analyses stratifying by cancer subsite (e.g. distal, proximal, colon, and rectal). This will require requesting access for restricted GWAS data ^36^.

We will explore conducting multi-variable MR (MVMR) ^37^ for significant findings (reaching an FDR corrected threshold of <5%) for which there is genetic overlap between instruments. We will use MVMR when we suspect this genetic overlap is more likely to reflect horizontal, as opposed to vertical, pleiotropy.

### Sensitivity analyses

For instrumental variable sets that use more than one genetic instrument, the analyses will be iteratively repeated, each time leaving out one SNP to test if there is a particular SNP that is driving results, which could be due to the outlying SNP being the only valid instrument or due to this SNP being horizontally pleiotropic.

We will apply “pleiotropy-robust” models as sensitivity analyses when sufficient independent genetic instruments are available (e.g. ≥ 10), given low statistical power of these models. These models include the weighted median ^38^, weighted mode ^39^ and MR Egger ^40^ regression and effect estimates from these methods can be compared to the IVW estimate obtained in initial analyses to evaluate if observed effects are possibly driven by horizontal pleiotropy. The weighted median can provide an unbiased causal estimate even when some of the genetic instruments used are invalid, the weighted mode effect estimate is more robust to horizontal pleiotropy, and the MR-Egger regression intercept can provide an indication of directional (i.e. unbalanced) pleiotropy. Whilst these sensitivity analyses relax certain assumptions of the IVW method, these models introduce new assumptions, so it is therefore important to use all three methods for comparison. If a consistent effect is observed across these three methods and the IVW estimate obtained from the main analyses, it is less likely that this finding is driven by violations of MR assumptions. To support these findings we will also use MR-PRESSO ^41^ to detect, and correct for, horizontal pleiotropy. This method assumes the majority of SNPs used as instruments are valid. 

For any exposures found to be associated with CRC risk, after applying an FDR correction, Steiger filtering will be used to explore the causal direction of SNP effects ^42^. The steiger_filtering function in the TwoSampleMR package will be used to evaluate if findings are driven by reverse causation, whereby the SNP used to instrument a molecular trait has an effect on CRC which in turn impacts variation in the molecular trait.

### Colocalisation

To determine if there is a shared causal variant between putative preventive agents and CRC risk, which is a requirement for inference of a causal effect of an agent on CRC risk, colocalisation will be conducted using the ‘coloc’ package in R ^43^. The default parameters will be used (e.g. the prior probabilities of the SNP being associated with the exposure, the outcome or both traits is specified as 1×°10 ^-4^, 1×°10 ^-4^ and 1×°10 ^-5^, respectively). As a sensitivity analysis we will use a more stringent threshold to test p12 (e.g. 1×°10 ^-6^). Bayes factor computation will be used to generate posterior probabilities (H0–H4) for the following 5 relationships between traits: (H0) neither trait has a causal variant in the region; (H1) only the molecular trait of interest has a causal variant in the region; (H2) only CRC risk has a causal variant in the region; (H3) both traits have associations within the genomic region but have different causal variants and (H4) both traits have the same causal variant. The posterior probability with the majority of support out of all configurations (e.g. a threshold of >0.50) will be used to evaluate evidence of a shared causal variant between preventive agents and CRC risk. Given that the posterior probability of colocalisation is on a continuum, we will classify PPH4 of 50% to 100% as providing a continuum of evidence in favour of shared causal variants (e.g. PPH4 ~ 50–80% as suggestive evidence for colocalisation, >80% as strong evidence for colocalisation). Colocalisation analyses will also be run using a method that allows for multiple causal variants, such as SuSiE ^44, 45^, to compare with the coloc results. Regional association plots will be generated to visualise results of colocalisation at the genetic region of interest using the LocusCompareR package ^46^.

### Data and software availability

This analysis will use publicly available summary statistics of CRC risk ( https://www.ebi.ac.uk/gwas/studies/GCST90255675) and molecular traits (either obtained through the GWAS catalogue, the IEU Open GWAS or directly from the published GWAS). For subsite-stratified analyses, access to this data will be requested through GECCO.

We will use the TwoSample MR package (v0.5.8) for MR analyses ( https://mrcieu.github.io/TwoSampleMR/), the ‘coloc’ package ( https://chr1swallace.github.io/coloc/) and SuSiE ( https://stephenslab.github.io/susieR/) for colocalisation analyses, and the LocusCompareR package ( https://github.com/boxiangliu/locuscomparer) to visualise regional genetic association data used in colocalisation analyses. All packages will be used in R.

## Study status

At the time of submission of this protocol for publication, preventive agents have been identified from the literature and we have identified the corresponding molecular trait for each agent. We have determined which molecular traits are instrumentable and, for those that are ( Table 2), we have begun to identify genetic instruments.

**Table 2.** Preventive agents identified in the literature to be associated with CRC risk reduction.

Preventive agent	Molecular trait
Dietary long-chain omega-3 polyunsaturated fatty acids	Circulating long-chain omega-3 polyunsaturated fatty acids
Dietary long-chain omega-6 polyunsaturated fatty acids	Circulating long-chain omega-6 polyunsaturated fatty acids
Dietary salicylic acid	Plasma salicylic acid levels
Dietary calcium	Serum calcium levels
Dietary vitamin D	Serum 25 hydroxyvitamin D
Dietary folate levels	Serum folate levels
Dietary selenium	Circulating selenium levels
Dietary vitamin A	Serum retinol levels
Dietary vitamin C	Plasma vitamin C levels
Dietary vitamin E	Circulating alpha-tocopherol levels
Dietary β carotene	Circulating β carotene levels
Dietary magnesium	Serum magnesium levels
Statins	Circulating LDL cholesterol levels (due to HMG-CoA reductase inhibition)
Guselkumab	Plasma IL23 protein levels
Antihypertensives	Serum ACE levels
	SBP (due to NCC inhibition)
	SBP (due to ADRB1 inhibition)
Testosterone	Circulating testosterone levels
Estradiol	Serum estradiol levels
Sex hormone binding globulin	Serum SHBG levels
Progesterone/17- hydroxyprogesterone	Circulating progesterone/17- hydroxyprogesterone levels

Abbreviations: CRC = colorectal cancer; LDL = low-density lipoprotein; HMG-CoA = 3-hydroxy-3-methylglutaryl coenzyme A; IL23 = interleukin 23; ACE = angiotensin-converting enzyme; SBP = systolic blood pressure; NCC = sodium chloride cotransporter; ADRB1 = β1 adrenoceptor; SHBG = sex hormone binding globulin. Preventive agents identified in the literature that had a corresponding molecular trait that was deemed instrumentable. These agents included dietary micronutrients, drugs and endogenous hormones. For each drug, the biological target of the drug was identified as it’s molecular trait, (e.g. the levels of the protein that the drug targets). For dietary micronutrients and endogenous hormones, molecular traits were identified that correspond to the agent (e.g. blood-based biomarkers for vitamin levels).

## Dissemination

The findings from this study, along with any changes in methodology, will be submitted for open access publication in a peer-reviewed journal upon completion of the study.

## Ethics and consent

The CRC GWAS used in this study complies with all relevant ethical regulations and has been approved by the South Central Ethics Committee (UK) (reference number 17/SC/0079). As the list of studies to be used to instrument the molecular traits is still being curated, details on the ethical compliance of these studies will be provided with the results of the analyses described here.

## Data Availability

No data are associated with this article.
