# Supplementary material for: Investigating the causal effect of previously reported therapeutic agents for colorectal cancer prevention: protocol for a Mendelian randomization analysis
Source: Wellcome Open Res. 2024 Jun 13;9:30. Originally published 2024 Feb 12. [Version 2] doi: 10.12688/wellcomeopenres.20861.2 (PMC11190651; doi:10.12688/wellcomeopenres.20861.2)
Supplement: Peer review history

# Reviewer report: Reviewer response for version 2

**DOI:** 10.21956/wellcomeopenres.24926.r86594

**Published:** 2024-06-21

**Authors:** Shuai Yuan

**Affiliations:** 1 Karolinska Institutet, Stockholm, Sweden

The authors did a good job in revision. I do not have further comments anymore.

Is the study design appropriate for the research question?

Partly

Is the rationale for, and objectives of, the study clearly described?

Yes

Are sufficient details of the methods provided to allow replication by others?

Yes

Are the datasets clearly presented in a useable and accessible format?

Not applicable

Reviewer Expertise:

NA

I confirm that I have read this submission and believe that I have an appropriate level of expertise to confirm that it is of an acceptable scientific standard.

# Reviewer report: Reviewer response for version 2

**DOI:** 10.21956/wellcomeopenres.24926.r86595

**Published:** 2024-06-20

**Authors:** Lanlan Chen, Wei Hao

**Affiliations:** 1 Jilin University, Jilin, China; 2 Jilin University (Ringgold ID: 12510), Changchun, Jilin, China

I have no further comments to make.

Is the study design appropriate for the research question?

Yes

Is the rationale for, and objectives of, the study clearly described?

Yes

Are sufficient details of the methods provided to allow replication by others?

Yes

Are the datasets clearly presented in a useable and accessible format?

Yes

Reviewer Expertise:

Genetic Epidemiology in Gastroenterology and Hepatology

We confirm that we have read this submission and believe that we have an appropriate level of expertise to confirm that it is of an acceptable scientific standard.

# Reviewer report: Reviewer response for version 1

**DOI:** 10.21956/wellcomeopenres.23083.r80182

**Published:** 2024-05-10

**Authors:** Shuai Yuan

**Affiliations:** 1 Karolinska Institutet, Stockholm, Sweden

This is a study protocol for an MR study to investigate the causal effect of previously reported therapeutic agents for colorectal cancer.

 Overall, the study is well-designed. The reviewer has a few comments: 

1. Exposure data and instrumental selection: maybe it is good to add that for protein IV selection, using cix index SNP as instruments is a widely acknowledged approach with high efficiency and low pleiotropy. 
2. It will be tricky to define “therapeutic” for CRC prevention. Including what types of therapeutic agents? Including dietary factors (like red meat intake, important for CRC). Should be specific when starting the SRs.
3. What formula was used to calculate R2? Is this calculable for traits not in SD unit based on summary-level data? 
4. Given many traits included as the exposures, what about the sample overlap between these exposure datasets and CRC GWAS?
5. Dietary intake does not always equal blood levels.

Is the study design appropriate for the research question?

Partly

Is the rationale for, and objectives of, the study clearly described?

Yes

Are sufficient details of the methods provided to allow replication by others?

Yes

Are the datasets clearly presented in a useable and accessible format?

Not applicable

Reviewer Expertise:

NA

I confirm that I have read this submission and believe that I have an appropriate level of expertise to confirm that it is of an acceptable scientific standard, however I have significant reservations, as outlined above.

# Response

**Published:** 2024-06-17

**Authors:** Ella Fryer

**Affiliations:** Population Health Sciences, University of Bristol, Bristol, England, UK

We thank the reviewer for their comments which we have addressed, point-by-point, below.

 1. Exposure data and instrumental selection: maybe it is good to add that for protein IV selection, using cix index SNP as instruments is a widely acknowledged approach with high efficiency and low pleiotropy.  

**Response: **We have now elaborated on the rationale for using cis-acting variants for select targets in the exposure data section, page 6.  

 Change in text: “For those molecular traits that are protein drug targets or biomarkers, SNPs will be selected that are located in or within proximity to genes that code for that protein (termed cis-variants). Cis-variants are expected to be specific to the target being instrumented which should minimise the likelihood of bias from horizontal pleiotropy. In addition, the use of cis-acting variants that influence protein targets of drugs examined would be expected to enhance instrument strength because protein expression is relatively proximal to germline genetic variants.”  

 2. It will be tricky to define “therapeutic” for CRC prevention. Including what types of therapeutic agents? Including dietary factors (like red meat intake, important for CRC). Should be specific when starting the SRs.

**Response:** We have chosen to only include agents for which the corresponding molecular trait can be pharmacologically targeted (e.g. circulating vitamin levels could be increased with supplements) or are pharmacological targets themselves (e.g. protein drug targets). We have clarified this in the study design section, page 4.  

 Change in text: “We are prioritising these types of agents as they can be modified through either supplementation (e.g. increasing vitamin levels) or pharmacological perturbation (e.g. inhibiting protein drug targets).”

 3. What formula was used to calculate R2? Is this calculable for traits not in SD unit based on summary-level data?

**Response: **We have updated the main analyses section, page 8-9, to show the formula that will be used to calculate R2. This formula takes into account the phenotypic variance of the trait and therefore does not require that traits are in SD units.   Change in text: “The proportion of variance explained in the instruments (R2) will be calculated using the following formula: 



 Where β is the effect size (beta coefficient) for a given SNP, MAF is the minor allele frequency, se(β) is the standard error of the effect size, and N is the sample size.  F-statistics will be calculated using the following formula:



 Where R2 is the proportion of variance explained in the instrument, N is the sample size”

 4. Given many traits included as the exposures, what about the sample overlap between these exposure datasets and CRC GWAS?  

**Response: **There will likely be some sample overlap between select exposure datasets and the CRC GWAS, especially given that the latter includes participants from the UK Biobank. We have now addressed this at the end of the outcome data section, page 7.  

 Change in text: “The summary data from this GWAS is available without UK Biobank data. Where there is sample overlap between the CRC GWAS and the exposure GWAS (i.e. those studies conducted exclusively in UK Biobank) and we have a significant finding from the main analyses after accounting for multiple testing (using a false discovery rate (FDR) correction of <5%), we will conduct a sensitivity analysis using CRC data with UK Biobank excluded. We will also compare the individual studies included in the CRC GWAS with all exposure datasets (i.e. beyond overlap of UK Biobank participants).

 Where there is a small-to-modest proportion of participant overlap between the studies included in the exposure data and the CRC data, we will quantify this overlap and, for any significant findings, we will repeat analyses using a more stringent F-statistic threshold (e.g. 30) to select genetic instruments. Sample overlap is only an issue if the genetic instruments used are weak (1, 2), therefore repeating analyses with a more stringent F-statistic threshold should minimise the likelihood of including weak instruments in analyses.”  

 5. Dietary intake does not always equal blood levels.

**Response:** We agree that blood-based biomarkers of micronutrients do not always correlate with dietary intake. When discussing potential implications of any significant findings in the main manuscript, we will be cautious of this limitation.

# Reviewer report: Reviewer response for version 1

**DOI:** 10.21956/wellcomeopenres.23083.r80184

**Published:** 2024-05-08

**Authors:** Lanlan Chen, Wei Hao

**Affiliations:** 1 Jilin University, Jilin, China; 2 Jilin University (Ringgold ID: 12510), Changchun, Jilin, China

Ella F et al. attempted to employ Mendelian randomization to investigate whether synthetic (e.g., approved medication) or natural (e.g., micronutrient, endogenous hormone) molecules could serve as potential targets for reducing the incidence of colorectal cancer according to this protocol. This protocol is rigorous and practical and I have only minor comments:

 1.    The priors in colocalization should be changed to test the robustness of the results (e.g., p1 = 1e-5, p2 = 1e-5, p12 = 1e-6 or p1 = 1e-4, p2 = 1e-4, p12 = 1e-6).

 2.    PPH4 > 0.5 indicated the colocalization, but it is not credible. Alternatively, the authors are recommended to focus on the results with PPH4 > 0.8 or even greater empirically.

 3.    Could bi-directional Mendelian randomization be employed to further assess reverse causation, especially for dietary nutrients and endogenous hormones?

Is the study design appropriate for the research question?

Yes

Is the rationale for, and objectives of, the study clearly described?

Yes

Are sufficient details of the methods provided to allow replication by others?

Yes

Are the datasets clearly presented in a useable and accessible format?

Yes

Reviewer Expertise:

Genetic Epidemiology in Gastroenterology and Hepatology

We confirm that we have read this submission and believe that we have an appropriate level of expertise to confirm that it is of an acceptable scientific standard, however we have significant reservations, as outlined above.

# Response

**Published:** 2024-06-17

**Authors:** Ella Fryer

**Affiliations:** Population Health Sciences, University of Bristol, Bristol, England, UK

We thank the reviewer for their comments which we have addressed, point-by-point, below.

 1.    The priors in colocalization should be changed to test the robustness of the results (e.g., p1 = 1e-5, p2 = 1e-5, p12 = 1e-6 or p1 = 1e-4, p2 = 1e-4, p12 = 1e-6).  

**Response:** We will still use the default settings initially and will repeat the analysis using a more stringent threshold for p12 (e.g. 1e-6). Please find this amendment in the colocalisation section, page 10.  

 Change in text: “The default parameters will be used (e.g. the prior probabilities of the SNP being associated with the exposure, the outcome or both traits is specified as 1×10 ^-4^, 1×10 ^-4^ and 1×10 ^-5^, respectively). As a sensitivity analysis we will use a more stringent threshold to test p12 (e.g. 1×10 ^-6^).”  

 2.    PPH4 > 0.5 indicated the colocalization, but it is not credible. Alternatively, the authors are recommended to focus on the results with PPH4 > 0.8 or even greater empirically.

**Response:** We agree that PPH4 >0.8 can be interpreted as strong evidence for colocalisation. However, PPH4 > 50% means that this configuration (of all configurations tested in colocalisation) has the highest posterior probability support. Given that the posterior probability of colocalisation is on a continuum, we will classify PPH4 > 50% to 100% as providing a continuum of evidence in favour of shared causal variants (e.g. PPH4 ~ 50-80% as suggestive evidence for colocalisation, >80% as strong evidence for colocalisation).  This has been updated in the colocalisation section, page 10.  

 Change in text: “The posterior probability with the majority of support out of all configurations (e.g. a threshold of >0.50) will be used to evaluate evidence of a shared causal variant between preventive agents and CRC risk. Given that the posterior probability of colocalisation is on a continuum, we will classify PPH4 of 50% to 100% as providing a continuum of evidence in favour of shared causal variants (e.g. PPH4 ~ 50-80% as suggestive evidence for colocalisation, >80% as strong evidence for colocalisation).” 

 3.    Could bi-directional Mendelian randomization be employed to further assess reverse causation, especially for dietary nutrients and endogenous hormones?

**Response:** The aim of this project is to appraise the evidence for potential preventive therapy agents in colorectal cancer development.  We therefore will not be examining whether CRC liability has an effect on these potential targets. However, we will explore whether individual SNPs included in instruments for targets reflect “reverse causation” (i.e. where the SNP has a primary effect on CRC risk and a secondary effect on the potential target) using Steiger filtering. Where there is evidence of misspecification of the causal direction of SNPs included in instruments, we will remove these SNPs from our analyses.**

 Section in text: “For any exposures found to be associated with CRC risk, after applying an FDR correction, Steiger filtering will be used to explore the causal direction of SNP effects (37). The steiger_filtering function in the TwoSampleMR package will be used to evaluate if findings are driven by reverse causation, whereby the SNP used to instrument a molecular trait has an effect on CRC which in turn impacts variation in the molecular trait.”

# Reviewer report: Reviewer response for version 1

**DOI:** 10.21956/wellcomeopenres.23083.r78000

**Published:** 2024-04-11

**Authors:** Yunyang DENG

**Affiliations:** 1 The Hong Kong Polytechnic University, Hong Kong, Hong Kong

I am pleased to review the manuscript, which aims to investigate whether several preventive agents are causally associated with colorectal cancer (CRC) using two-sample Mendelian randomization (MR) analyses. Here are some comments to the authors:

- Data sources: Although the exact data sources of exposures cannot be decided yet, I can imagine that there might be some sample overlap. Therefore, relevant descriptions and discussion should be added.
- Methods: “Of these variants, only those that are independent (r2<0.01) will be included as instrumental variables in the primary analyses.” What is the evidence for the LD-r2 threshold? As far as I know, though 0.01 is a common choice, some previous MR studies used 0.001. I think this threshold should be decided based on the number of SNPs for the exposure(s).
- Statistical analysis: Sometimes, these factors may interact with each other. Therefore, will the authors consider performing multivariable MR analyses?
- Statistical analysis: MR-PRESSO tests should be conducted to partially test the horizontal pleiotropy.
- Statistical analysis: The authors should build leave-one-out plots and forest plots to see the independent effect of each SNP and to detect any potential outlier SNPs that significantly affected the results.

Is the study design appropriate for the research question?

Yes

Is the rationale for, and objectives of, the study clearly described?

Yes

Are sufficient details of the methods provided to allow replication by others?

Partly

Are the datasets clearly presented in a useable and accessible format?

Partly

Reviewer Expertise:

Public health; Epidemiology; Mendelian randomization; Cancer; Non-communicable diseases.

I confirm that I have read this submission and believe that I have an appropriate level of expertise to confirm that it is of an acceptable scientific standard, however I have significant reservations, as outlined above.

# Response

**Published:** 2024-06-17

**Authors:** Ella Fryer

**Affiliations:** Population Health Sciences, University of Bristol, Bristol, England, UK

We thank the reviewer for their comments which we have addressed, point-by-point, below.

 Data sources: Although the exact data sources of exposures cannot be decided yet, I can imagine that there might be some sample overlap. Therefore, relevant descriptions and discussion should be added.  

**Response:** We expect that there will be small-to-modest sample overlap between select exposure GWAS and the CRC susceptibility GWAS given that the latter sourced some participants from the UK Biobank (cases=4800, control=20289). We have now addressed this at the end of the outcome data section (page 7):  

 Change in text: “The summary data from this GWAS is available without UK Biobank data. Where there is sample overlap between the CRC GWAS and the exposure GWAS (i.e. those studies conducted exclusively in UK Biobank) and we have a significant finding from the main analyses after accounting for multiple testing (using a false discovery rate (FDR) correction of <5%), we will conduct a sensitivity analysis using CRC data with UK Biobank excluded. We will also compare the individual studies included in the CRC GWAS with all exposure datasets (i.e. beyond overlap of UK Biobank participants). Where there is a small-to-modest proportion of participant overlap between the studies included in the exposure data and the CRC data, we will quantify this overlap and, for any significant findings, we will repeat analyses using a more stringent F-statistic threshold (e.g. 30) to select genetic instruments. Sample overlap is only an issue if the genetic instruments used are weak (1, 2), therefore repeating analyses with a more stringent F-statistic threshold should minimise the likelihood of including weak instruments in analyses.”  

 Methods: “Of these variants, only those that are independent (r2<0.01) will be included as instrumental variables in the primary analyses.” What is the evidence for the LD-r2 threshold? As far as I know, though 0.01 is a common choice, some previous MR studies used 0.001. I think this threshold should be decided based on the number of SNPs for the exposure(s).

**Response: **We agree with the reviewer that r ^2^<0.001 is often used in MR studies. Given that this threshold is more stringent (i.e. SNPs are less likely to be in low LD as compared to using a r2<0.01 threshold) we have now updated the threshold in our manuscript to 0.001. Any deviations from this threshold (e.g. to allow more SNPs, that may be weakly correlated, to be included as genetic instruments for protein drug targets) will be justified in the main manuscript. As can now be seen on page 6:  

 Change in text: “Of these variants, only those that are independent (r 2<0.001) will be included as instrumental variables in the primary analyses. For protein drug targets and biomarkers, we will increase the number of SNPs in our genetic instrument by permitting SNPs to be in weak linkage disequilibrium (e.g. r 2<0.10). We will account for this by generating a correlation matrix using a random subset of 10,000 individuals of white British ancestry in the UK Biobank as reference panel and using a generalised IVW method that can account for correlated instruments (1).”  

 Statistical analysis: Sometimes, these factors may interact with each other. Therefore, will the authors consider performing multivariable MR analyses?

**Response:** For significant findings (reaching an FDR corrected threshold of <5%) where there is genetic overlap between instruments, and we believe this could lead to horizontal pleiotropy (e.g. in the case of omega-3 and omega-6 polyunsaturated fatty acids), we will conduct MVMR. Please see addition to end of Main analyses section, page 9.  

 Change in text: “We will explore conducting multivariable MR (MVMR) (4) for significant findings (reaching an FDR corrected threshold of <5%) for which there is genetic overlap between instruments. We will use MVMR when we suspect this genetic overlap is more likely to reflect horizontal, as opposed to vertical, pleiotropy.”

 Statistical analysis: MR-PRESSO tests should be conducted to partially test the horizontal pleiotropy.  

**Response:** We have added this in the sensitivity analyses section, page 9.    Change in text: “To support these findings we will also use MR-PRESSO (5) to detect, and correct for, horizontal pleiotropy. This method assumes that the majority of SNPs used as instruments are valid.”  

 Statistical analysis: The authors should build leave-one-out plots and forest plots to see the independent effect of each SNP and to detect any potential outlier SNPs that significantly affected the results.  

**Response:** We will be conducting leave-one-out analyses (please see first paragraph of sensitivity analyses section); however, for some traits there are a large number of SNPs (e.g. >100) available to instrument that trait so any plots generated would be difficult to interpret. We will therefore conduct leave-one-out analyses but not generate plots where there are large numbers of SNPs in instruments. If removing a single SNP means that the recalculated OR (95% CI) does not overlap with the results from the primary analysis, this outlying SNP could be a plausible outlier and we may consider removing it from the primary analysis if suspected to be horizontally pleiotropic.
